# Place of death and other factors associated with unnatural mortality in patients with serious mental disorders: population-based retrospective cohort study

**DOI:** 10.1192/bjo.2019.5

**Published:** 2019-03-04

**Authors:** Rebecca Wilson, Fiona Gaughran, Tara Whitburn, Irene J. Higginson, Wei Gao

**Affiliations:** Research Associate, Cicely Saunders Institute of Palliative Care, Policy & Rehabilitation, Florence Nightingale Faculty of Nursing, Midwifery & Palliative Care, King's College London, UK; Lead Consultant/Senior Lecturer, Psychosis Studies Department, Institute of Psychiatry, Psychology and Neuroscience, King's College London; and National Psychosis Unit, South London and Maudsley NHS Foundation Trust, UK; Consultant in Palliative Medicine, Barts Health NHS Trust, Macmillan Palliative Care Team, St Bartholomew's Hospital, UK; Head of Department, Head of Division and Director of Cicely Saunders Institute, Cicely Saunders Institute of Palliative Care, Policy and Rehabilitation, Florence Nightingale Faculty of Nursing, Midwifery and Palliative Care, King's College London, UK; Reader in Statistics and Epidemiology, Cicely Saunders Institute of Palliative Care, Policy and Rehabilitation, Florence Nightingale Faculty of Nursing, Midwifery & Palliative Care, King's College London, UK

**Keywords:** Serious mental disorder, unnatural cause of death, place of death, routine data, mortality

## Abstract

**Background:**

Patients with serious mental disorders have poorer healthcare outcomes at the end of life and are at greater risk of dying from unnatural causes.

**Aims:**

To explore place of death and demographic and clinical correlates of unnatural causes of death in patients with serious mental disorders.

**Method:**

Routinely collected patient data were used to explore bivariate and adjusted associations between covariates and natural/unnatural cause of death.

**Results:**

In multivariable analysis (*n* = 1029), dying at home (odds ratio (OR) = 1.87, 95% CI 1.03–3.40), ‘other’ locations (OR = 16.50, 95% CI 7.57–36.00), younger age (OR = 17.26, 95% CI 8.28–36.00) and a diagnosis other than schizophrenia spectrum disorder (OR = 1.69, 95% CI 1.04–2.73) were correlates of unnatural cause of death.

**Conclusions:**

Deaths from unnatural causes were high and more likely to occur at home and non-healthcare settings. Unnatural causes of death were higher in younger patients with non-schizophrenia spectrum disorder diagnoses.

**Declaration of interest:**

F.G. has received support or honoraria for CME, advisory work and lectures from Bristol-Myers Squibb, Janssen, Lundbeck, Otsuka, Roche, and Sunovion, and has a family member with professional links to Lilly and GSK, including shares.

Patients with serious mental disorders have shorter lives compared with the general population.^1^^–^^6^ Although the gap in mortality is largely because of increased risk of premature death from natural causes,^3^^,^^7^ rates of deaths from unnatural (or external) causes are also higher in patients with serious mental disorders than in the general population. Unnatural causes of death include suicides, homicides and accidental deaths.^8^ In the UK general population, deaths from unnatural causes represent 3.5% of all deaths,^9^ whereas, in patients with serious mental disorders, proportions of unnatural deaths range from 17.5% to 25% of deaths,^5^^,^^10^^,^^11^ depending on the population included.

Previous research has identified various correlates of death from unnatural causes in patients with serious mental disorders, although studies differ on diagnostic inclusion criteria. Patients who die from unnatural causes are likely to be younger age,^12^^–^^14^ male,^3^^,^^12^^,^^15^^,^^16^ have a psychiatric diagnosis other than schizophrenia spectrum disorder (SSD)^11^^,^^17^ and have been recently discharged from a psychiatric hospital.^12^ However, it should be noted that many of these studies have focused on identifying the correlates of suicide,^11^^–^^13^^,^^16^^,^^17^ rather than deaths from unnatural causes collectively.

## Health care experiences of people with serious mental disorders

Patients with serious mental disorders have poorer access to and provision of physical healthcare.^7^^,^^18^ However, it is also reported that serious mental disorders are associated with more acute service use, particularly accident and emergency (A&E) visits and hospital admissions.^19^ Previous research has shown that patients have a high risk of avoidable mortality following discharge from both acute and psychiatric hospitals.^12^^,^^20^

Place of death is often used as an indicator of the quality of care at the end of life.^21^^,^^22^ Several factors related to place of death have been identified,^23^ including some that may be pertinent to patients with serious mental disorders: healthcare input, social support and functional status. There is sparse research exploring place of death in this underserved population. One retrospective cohort study reported that the most common place of death in a study of patients with schizophrenia was hospital, followed by nursing homes.^24^ Suicides from within this group predominantly occurred in the home (47.5%) and around a third occurred in hospital (33.3%). These proportions were similar to a matched cohort of patients without a schizophrenia diagnosis.

## Study aim

Deaths from unnatural causes are preventable. Identifying factors associated with these deaths in a potentially vulnerable and underserved population may illustrate groups of individuals who are at risk or have a greater likelihood of death from unnatural causes. This study aimed to assess place of death in a sample of patients with serious mental disorders who died from natural and unnatural causes and to explore associations between a number of health and social factors and deaths from unnatural causes.

## Method

This was a retrospective cohort study using routinely collected data from the Clinical Record Interactive Search (CRIS) system, developed by the South London and Maudsley (SLAM) Biomedical Research Centre Case Register. SLAM is a secondary mental healthcare provider whose patients reside in south-east London and uses electronic health records for routine patient care. These data (collected from patients who gave consent for their data to be included in CRIS) were linked with mortality data from the Office for National Statistics and Hospital Episodes Statistics. Through CRIS, these electronic records are available for secondary data analysis in research.^25^^,^^26^ The research ethics approval, granted by the Oxfordshire Research Ethics Committee C (reference 08/H0606/71 + 5), allows the secondary analysis of CRIS-derived data. Ethical approval for this study was received from the CRIS oversight committee.

Patients were included in the present study if they died between 1 April 2007 and 31 March 2013, had complete data for cause of death and place of death and had a diagnosis of serious mental illness (as identified by ICD-10 codes: schizophrenia (F20), schizoaffective disorder (F25) or bipolar affective disorder (F31)) or other serious mental disorders (substance use disorders (F10–19), depressive episode (F32) or recurrent depressive disorder (F33)).^27^

### Measures

#### Outcome measure

Cause of death was dichotomised as a binary measure of natural/unnatural deaths (0, natural cause of death; 1, unnatural cause of death). Unnatural deaths included the following underlying causes of death: injuries (S00–T19), burns/frostbite (T20–T35), poisoning (T36–T78), transport accidents (V01–V99), falls/other accidents (W00–X59), intentional self-harm (X60–X84, Y10–Y34), assault (X85–Y09 & U50.9) and complications of care and other causes (T79–T98, Y35–36, Y40–Y98).^9^ All other causes of death were coded as natural causes of death.

### Explanatory variables

#### Demographic factors

Place of death was categorised as care homes, patients' homes, hospices, hospitals and ‘other’ locations – which included outdoor spaces, home addresses other than the patient's and other institutional buildings. Other demographic factors included age at death, gender, ethnicity (dichotomised as White British/Irish compared with all other ethnicities), area-level deprivation (measured using area-level deprivation quintiles (1, high deprivation; 5, low deprivation) defined by the Index of Multiple Deprivation at lower layer super output area level)^28^ and marital status.

#### Clinical factors

Risk status was measured using a binary indicator denoting patients who were identified as ‘at risk’, compared with not at risk (a brief risk assessment by SLAM healthcare professionals is a compulsory target for all patients who access SLAM, those flagged as ‘at risk’ then received a full risk assessment). Primary mental disorder diagnosis was recorded by SLAM professionals at each contact, thus many patients have multiple primary diagnosis. Where patients had any diagnosis of SSD, this was recorded as their primary diagnosis and a binary variable denoting SSD/other mental disorder was created. A categorical measure of healthcare in the last month of life indicated whether patients had been admitted to hospital (either general or psychiatric), had visited A&E, both or neither.

### Statistical analysis

Ethnicity, marital status and area-level deprivation had missing data. Missing data were imputed using chained equations. Five imputations were created and estimates were combined using Rubin's rules.^29^

Descriptive statistics were explored for patients who died from a natural or unnatural cause. Bivariate associations between explanatory variables (demographic and clinical factors) and the outcome variable (natural/unnatural cause of death) were assessed using logistic regression. Factors bivariately associated with the outcome variable (*P* < 0.05) were included in a fully adjusted multivariable logistic regression model. Although we refer to ‘explanatory’ and ‘outcome’ variables, the analysis does not infer causality and the study was exploratory, with the aim of assessing the different health and social factors associated with patients who died from natural and unnatural causes.

Sensitivity analyses confirmed no meaningful difference between complete case models and imputed models. Imputed models were used to preserve the sample size. All analysis was performed using Stata 14.

## Results

The study included 1029 patients who met the eligibility criteria. Descriptive statistics, stratified by natural/unnatural cause of death are presented in Table 1. Of the 1029 patients included in the analysis, 86.5% (*n* = 890) died from natural causes and 13.5% (*n* = 139) died from unnatural causes.

**Table 1:** Patients' descriptive statistics stratified by natural/unnatural cause of death^a^

	Natural cause of death (*n* = 890)	Unnatural cause of death (*n* = 139)
*Demographic factors*		
Place of death, *n* (%)		
Care home	93 (10.4)	1 (0.7)
Home	278 (31.2)	55 (39.6)
Hospice	30 (3.4)	0 (0.0)
Hospital	469 (52.7)	33 (23.7)
Other	20 (2.2)	50 (36.0)
Age at death, years		
18–44, *n* (%)	102 (11.5)	87 (62.6)
45–64, *n* (%)	295 (33.1)	39 (28.1)
≥65, *n* (%)	493 (55.4)	13 (9.4)
Mean (s.d.)	65.8 (15.4)	42.6 (15.3)
Gender, *n* (%) male	461 (51.8)	94 (67.6)
Ethnicity (imputed proportions)		
White British/Irish	62.3	57.1
Other ethnicities	37.7	42.9
IMD quintiles (imputed proportions)		
1 (highest deprivation)	35.6	38.1
2	36.3	36.4
3	18.6	16.7
4	4.7	5.0
5 (lowest deprivation)	4.9	3.7
Marital status (imputed proportions)		
Married/civil partner/cohabiting	14.6	8.9
Separated/divorced/widowed	30.0	12.1
* *Single	55.4	79.0
*Clinical factors*		
Identified as at risk, *n* (%)	332 (37.3)	67 (48.2)
Primary diagnosis, *n* (%)		
SSD	655 (73.6)	88 (63.3)
Non-SSD	235 (26.4)	51 (36.7)
Healthcare in last month of life, *n* (%)		
No admission and no A&E	421 (47.3)	98 (70.5)
Admitted and no A&E	63 (7.1)	4 (2.9)
A&E and not admitted	71 (8.0)	15 (10.8)
A&E and admitted	335 (37.6)	22 (15.8)

IMD, Index of Multiple Deprivation; SSD, schizophrenia spectrum disorder; A&E, accident and emergency.

a. Numbers rounded to one decimal place; where column % totals do not equal 100%, this is because of rounding.

Descriptive results demonstrated that, of those who died from unnatural causes, the largest proportion of deaths occurred in the home (39.6%; followed by other locations, 36%), whereas the majority of deaths from natural causes were in hospital (52.7%). Compared with patients who died from natural causes, patients who died from unnatural causes were younger (mean age  42.6 years, compared with mean age = 65.8 years) and predominantly male (67.6% compared with 51.8%). More were of ethnic backgrounds other than White British/Irish (42.9% compared with 37.7%) and comparable numbers lived in the most deprived quintile (38.1% compared with 35.6%).

Compared with patients who died from natural causes, those who died from unnatural causes were also predominantly single (79% compared with 55.4%), a higher proportion had been identified as being at risk (48.2% compared with 37.3% of patients who died from natural causes) and a greater proportion had a primary non-SSD psychiatric diagnosis (36.7% compared with 26.4%). These non-SSD diagnoses included affective psychosis (21.6% of deaths from unnatural causes), mood disorders/neuroses (9.4%) and organic/substance use disorders (5.8%). The majority of patients who died from unnatural causes neither visited A&E nor were admitted to a (general/psychiatric) hospital in the last month of life (70.5%), whereas this was the case for less than half of patients who died from natural causes (47.3%).

As a result of the low numbers of deaths in hospices and care homes (particularly in patients who died from unnatural causes), care home, hospice and hospital were collapsed into a single category denoting ‘institutional care settings’ in the place of death variable. In bivariate analyses (Table 2), dying at home or in other locations (compared with in a care setting), younger age, being male, being single compared with married/in a civil partnership/cohabiting, being identified as at risk, having a non-SSD primary diagnosis, neither visiting A&E nor being admitted to hospital in the last month of life (compared with both visiting A&E and being admitted) and visiting A&E but not being admitted were all significantly associated with an unnatural cause of death.

**Table 2:** Unadjusted and multiply adjusted odds ratios (95% CI) for demographic and clinical factors and unnatural cause of death in the sample (*n* = 1029)

	Unadjusted OR (95% CI)	Multiply adjusted OR (95% CI)
Place of death		
Care home/hospital/hospice	Reference	Reference
Home	3.44 (2.19–5.41)*	1.87 (1.03–3.40)*
Other	43.53 (23.34–81.18)*	16.50 (7.57–36.00)*
Age at death, years		
18–44	32.35 (17.39–60.16)*	17.26 (8.28–36.00)*
45–64	5.01 (2.63–9.55)*	3.55 (1.77–7.13)*
≥65 years	Reference	Reference
Gender: male compared with female	1.94 (1.33–2.84)*	0.99 (0.61–1.61)
Index of Multiple Deprivation quintiles		
1	Reference	Reference
2	0.93 (0.61–1.41)	–
3	0.82 (0.48–1.40)	–
4	0.98 (0.42–2.30)	–
5	0.72 (0.27–1.95)	–
Marital status		
Married/civil partner/cohabiting	Reference	Reference
Separated/divorced/widowed	0.67 (0.29–1.51)	0.80 (0.30–2.13)
Single	2.33 (1.22–4.46)*	0.79 (0.35–1.76)
Ethnicity: others compared with White British/Irish reference category	1.12 (0.94–1.35)	–
At risk: compared with not at risk	1.56 (1.09–2.24)*	1.19 (0.76–1.87)
Primary diagnosis: non-SSD compared with SSD	1.69 (1.29–2.20)*	1.69 (1.04–2.73)*
Healthcare in last month of life		
A&E and admission	Reference	Reference
No A&E and not admitted	3.54 (2.18–5.75)*	1.14 (0.59–2.21)
Admitted and no A&E	0.97 (0.32–2.90)	0.72 (0.22–2.35)
A&E and not admitted	3.22 (1.59–6.51)*	1.76 (0.78–3.96)

SSD, schizophrenia spectrum disorder; A&E, accident and emergency.

* *P* < 0.05.

In a multiply adjusted model, dying at home or other location, younger age and having a non-SSD diagnosis remained significant correlates of unnatural cause of death following all adjustments. Associations with gender, marital status, risk status and healthcare use were all attenuated.

## Discussion

### Place of death

Descriptive analyses showed substantial differences in the place of death for patients who died from natural and unnatural causes; deaths from unnatural causes were higher at home and in other locations, whereas deaths from natural causes were higher in healthcare settings. In further analysis, place of death was significantly associated with unnatural cause of death in the fully adjusted model; unnatural deaths were more likely to occur in the home or in ‘other’ locations than in healthcare settings after adjusting for other relevant factors such as age, gender and healthcare access in the last month of life. Moreover, these data demonstrate that place of death cannot be used in the same way that it is for other populations as a proxy measure for quality of end-of-life care across a population of patients with serious mental disorders. As deaths from unnatural causes are higher in this population than in the general population, this should be taken into consideration when investigating place of death in patients with serious mental disorders and other mental disorders.

### Diagnosis

In addition to place of death, the results demonstrated that younger age and a diagnosis other than SSD were strong correlates of dying from unnatural causes and attenuated associations between being male, single and at risk and unnatural death. These results are in line with existing research, as younger age was previously reported to be associated with increased likelihood of death by unnatural causes.^12^^–^^14^ By including all unnatural causes of death, the present study expanded on the results of Lopez-Morinigo *et al*^12^ and Melle *et al*,^13^ which included only deaths by suicide. Exisiting literature has also explored psychiatric diagnosis as a correlate of unnatural causes of death, particularly suicide. Lopez-Morinigo *et al*^17^ highlighted suicide risk factors in patients with serious mental disorders other than SSD and Hiroeh *et al*^11^ reported that suicide risk was greatest for patients with substance use disorders, affective disorders and personality disorders. However, it should be considered that patients with SSD may have a different experience of care pathways to other diagnoses, because of diagnostic biases that might lead to prioritisation and preferential care provision from psychiatric services. Patients with SSD may be more likely to receive intervention from healthcare providers. Thus, it is likely that there are many other contributing factors to the association between psychiatric diagnosis and cause of death and we cannot conclude that a non-SSD diagnosis is predictive of unnatural cause of death.

### Hospital care in the last month of life

Receiving no hospital care in the last month of life (neither visiting A&E nor being admitted to general/psychiatric hospital) and visiting A&E but not being admitted to hospital were both bivariately associated with death from unnatural causes, but these associations were attenuated by place of death in the adjusted model. In further inspection of the data (see supplementary Table 1 available at https://doi.org/10.1192/bjo.2019.5), healthcare acutely accessed in the last month of life was closely related to place of death; of the 510 patients who acutely accessed healthcare in the last month of life (visited A&E and/or were admitted to hospital), 79.2% (*n* = 404) died in hospital (93.3% from natural causes). As most people who died in hospital (whether admitted before or during the last month of life) died from natural causes (*n* = 469, 93.4% of the total 502 hospital deaths), it cannot be concluded that not accessing acute healthcare in the last month of life is directly associated with the increased likelihood of dying from unnatural causes. The results are more likely to suggest that patients (with serious mental disorders) with physical health problems are likely to either visit A&E or be admitted to hospital and then die in hospital. Further research is needed to explore the relationship between healthcare use at the end of life, cause of death and place of death.

### Risk assessments

Previous literature has explored the role of risk assessments, using similar data, and showed that patients with SSD were more like to have had a full risk assessment than patients with a non-SSD diagnosis.^17^ The present study found that, after adjusting for other covariates, there was not a significant association between receiving a full risk assessment, thereby being identified as ‘at risk’ by staff, and death from unnatural causes. These results are consistent with the conclusion drawn by Lopez-Morinigo *et al*,^17^ in that, we did not observe an association between contact with a healthcare professional and receiving a risk assessment and likelihood of an unnatural death.

### Proportion of unnatural deaths

Deaths because of unnatural causes were higher in this sample of patients with serious mental disorders and other mental disorders (13.5%) than for the general population (3.5%).^9^ However, the proportion of unnatural deaths from this study was lower than rates reported by previous studies; rates of unnatural causes have been reported between 17.5% and 25% of deaths in patient with serious mental disorders.^5^^,^^10^^,^^11^ This may be because the present sample came from patients who were under the care of a mental healthcare trust at the time of death and thus were more likely to be receiving psychiatric care. Other studies reporting higher rates of deaths from unnatural causes included patients who had ever had a psychiatric hospital admission^11^ or just included patients with schizophrenia.^10^ A meta-analysis^5^ provided a more conservative prevalence rate (17.5%), closer to the one observed in these data.

### Strengths and limitations

Strengths of this study include the use of linked data, making use of routinely collected data in research. Missing data were minimal, demonstrating the quality and richness of the data. Place of death and care at end of life are particularly understudied in this patient group, despite the increased mortality risk. This study adds to this knowledge gap. One limitation of this exploratory study is that the results do not infer causality and associations should not be interpreted as directional. This study utilised data from south London only. Although SLAM is the largest mental healthcare provider in Europe with a population of 1.1 million patients,^25^ this is a limited geographic range and thus results may not be generalisable. As a result of the small numbers in the group of patients that died from unnatural causes, some categorical variables presented small cell counts and therefore some variables (place of death, primary diagnosis) had to be collapsed, limiting the level of detail the results were able to assess. Future work could aim to utilise a large sample.

### Implications

This work preludes further work into end-of-life care and place of death in patients with serious mental disorders. The results have shown that place of death differs by natural and unnatural cause of death, therefore future work on place of death should be stratified by, or focus on either natural or unnatural causes of death. In line with previous research, this study found younger age was associated with death from unnatural causes and that there was an excess of unnatural mortality at home as well as other locations (outdoor spaces, home addresses other than the patient's and other institutional buildings). One implication for clinical practice and policy is to highlight a group of patients potentially at greater risk of death from unnatural causes; younger patients outside of healthcare settings. This study added to previous research by including unnatural causes other than suicide; younger patients are equally at greater risk of dying from unnatural causes other than suicide. Given that these deaths are potentially preventable, these results would suggest that younger patients with serious mental disorders may require extra support from health and social care providers. Any interventions to reduce the risk of suicide or deaths from unnatural causes should target these patients. Further research should explore the potential mechanisms by which this association might operate.

In conclusion, patients with serious mental disorders have higher rates of death from unnatural causes than the general population and these deaths are more likely to occur in the home or other non-healthcare settings. Our results suggest that younger patients and those with diagnoses other than SSD are less likely to die from natural causes, although the mechanisms behind these associations are unknown. Furthermore, patients who die from unnatural causes are less likely to have been admitted to hospital prior to death. This study has also demonstrated the benefits of using routinely collected clinical data in research and will inform further research in end-of-life care for patients with serious mental disorders.

## Supplementary material

For supplementary material accompanying this paper visit http://dx.doi.org/10.1192/bjo.2019.5. click here to view supplementary material

## References

[1] Brown S, Kim M, Mitchell C, Inskip H. Twenty-five year mortality of a community cohort with schizophrenia. Br J Psychiatry 2010; 196: 116–21. 20118455 10.1192/bjp.bp.109.067512 PMC4560167

[2] Kisely S, Crowe E, Lawrence D. Cancer-related mortality in people with mental illness. JAMA Psychiatry 2013; 70:209–17. 23247556 10.1001/jamapsychiatry.2013.278

[3] Lawrence D, Hancock KJ, Kisely S. The gap in life expectancy from preventable physical illness in psychiatric patients in Western Australia: retrospective analysis of population based registers. BMJ 2013; 346: f2539. 23694688 10.1136/bmj.f2539 PMC3660620

[4] Ösby U, Westman J, Hällgren J, Gissler M. Mortality trends in cardiovascular causes in schizophrenia, bipolar and unipolar mood disorder in Sweden 1987–2010. Eur J Public Health 2016; 26: 867–71. 26748100 10.1093/eurpub/ckv245 PMC5054269

[5] Reisinger Walker E, McGee RE, Druss BG. Mortality in mental disorders and global disease burden implications: a systematic review and meta-analysis. JAMA Psychiatry 2015; 72: 334–41. 25671328 10.1001/jamapsychiatry.2014.2502 PMC4461039

[6] Viron MJ, Stern TA. The impact of serious mental illness on health and healthcare. Psychosomatics 2010; 51: 458–65. 21051676 10.1176/appi.psy.51.6.458

[7] Moore S, Shiers D, Daly B, Mitchell AJ, Gaughran F. Promoting physical health for people with schizophrenia by reducing disparities in medical and dental care. Acta Psychiatr Scand 2015; 132: 109–21. 25958971 10.1111/acps.12431

[8] Sonderman JS, Munro HM, Blot WJ, Tarone RE, McLaughlin JK. Suicides, homicides, accidents, and other external causes of death among blacks and whites in the southern community cohort study. Niederkrotenthaler T, editor PLoS One 2014; 9: e114852. 25486418 10.1371/journal.pone.0114852 PMC4259484

[9] National End of Life Care Intelligence Network. External Causes of Death. National End of Life Care Intelligence Network, 2011.

[10] Brown S, Inskip H, Barraclough B. Causes of the excess mortality of schizophrenia. Br J Psychiatry 2000; 177: 212–7. 11040880 10.1192/bjp.177.3.212

[11] Hiroeh U, Appleby L, Mortensen PB, Dunn G. Death by homicide, suicide, and other unnatural causes in people with mental illness: a population-based study. Lancet 2001; 358: 2110–2. 11784624 10.1016/S0140-6736(01)07216-6

[12] Lopez-Morinigo J-D, Ayesa-Arriola R, Torres-Romano B, Fernandes AC, Shetty H, Broadbent M, Risk assessment and suicide by patients with schizophrenia in secondary mental healthcare: a case–control study. BMJ Open 2016; 6: e011929. 10.1136/bmjopen-2016-011929 PMC5051464 27678536

[13] Melle I, Olav Johannesen J, Haahr UH, ten Velden Hegelstad W, Joa I, Langeveld J, Causes and predictors of premature death in first-episode schizophrenia spectrum disorders. World Psychiatry 2017; 16: 217–8. 28498600 10.1002/wps.20431 PMC5428196

[14] Meloni D, Miccinesi G, Bencini A, Conte M, Crocetti E, Zappa M, Mortality among discharged psychiatric patients in Florence, Italy. Psychiatr Serv 2006; 57: 1474–81. 17035568 10.1176/ps.2006.57.10.1474

[15] Brown S. Excess mortality of schizophrenia. A meta-analysis. Br J Psychiatry 1997; 171: 502–8. 9519087 10.1192/bjp.171.6.502

[16] Dutta R, Murray RM, Allardyce J, Jones PB, Boydell J. Early risk factors for suicide in an epidemiological first episode psychosis cohort. Schizophr Res 2011; 126: 11–9. 21183318 10.1016/j.schres.2010.11.021

[17] Lopez-Morinigo JD, Fernandes AC, Chang CK, Hayes RD, Broadbent M, Stewart R, Suicide completion in secondary mental healthcare: a comparison study between schizophrenia spectrum disorders and all other diagnoses. BMC Psychiatry 2014; 14: 213. 25085220 10.1186/s12888-014-0213-z PMC4149212

[18] Ringen PA, Engh JA, Birkenaes AB, Dieset I, Andreassen OA. Increased mortality in schizophrenia due to cardiovascular disease - a non-systematic review of epidemiology, possible causes and interventions. Front Psychiatry 2014; 5: 137. 25309466 10.3389/fpsyt.2014.00137 PMC4175996

[19] Lally J, Wong YL, Shetty H, Patel A, Srivastava V, Broadbent MTM, Acute hospital service utilization by inpatients in psychiatric hospitals. Gen Hosp Psychiatry 2015; 37: 577–80. 26319481 10.1016/j.genhosppsych.2015.07.006

[20] Hoang U, Goldacre MJ, Stewart R. Avoidable mortality in people with schizophrenia or bipolar disorder in England. Acta Psychiatr Scand 2013; 127: 195–201. 23216065 10.1111/acps.12045

[21] Sleeman KE, Ho YK, Verne J, Glickman M, Silber E, Gao W, Place of death, and its relation with underlying cause of death, in Parkinson's disease, motor neurone disease, and multiple sclerosis: a population-based study. Palliat Med 2013; 27: 840–6. 23737036 10.1177/0269216313490436

[22] De Roo ML, Miccinesi G, Onwuteaka-Philipsen BD, Van Den Noortgate N, Van Den Block L, Bonacchi A, Actual and preferred place of death of home-dwelling patients in four European countries: making sense of quality indicators. PLoS One 2014; 9: e93762. 24714736 10.1371/journal.pone.0093762 PMC3979710

[23] Gomes B, Higginson IJ. Factors influencing death at home in terminally ill patients with cancer: systematic review. BMJ 2006; 332: 515–21. 16467346 10.1136/bmj.38740.614954.55 PMC1388126

[24] Martens PJ, Chochinov HM, Prior HJ. Where and how people with schizophrenia die: a population-based, matched cohort study in Manitoba, Canada. J Clin Psychiatry 2013; 74: 551–7. 10.4088/JCP.12m08234 23842025

[25] Stewart R, Soremekun M, Perera G, Broadbent M, Callard F, Denis M, The South London and Maudsley NHS Foundation Trust Biomedical Research Centre (SLAM BRC) case register: development and descriptive data. BMC Psychiatry 2009; 9: 1–12. 19674459 10.1186/1471-244X-9-51 PMC2736946

[26] Perera G, Broadbent M, Callard F, Chang C-K, Downs J, Dutta R, Cohort profile of the South London and Maudsley NHS Foundation Trust Biomedical Research Centre (SLaM BRC) Case Register: current status and recent enhancement of an Electronic Mental Health Record-derived data resource. BMJ Open 2016; 6: e008721. 10.1136/bmjopen-2015-008721 PMC4785292 26932138

[27] World Health Organization. The ICD-10 Classification of Mental and Behavioural Disorders: Clinical Descriptions and Diagnostic Guidelines. WHO, 1992.

[28] Payne R, Abel G. UK Indices of Multiple Deprivation - a Way to Make Comparisons Across Constituent Countries. Health Statistics Quarterly. Office for National Statistics, 2012.

[29] Rubin DB, Schenker N. Multiple imputation in health-care databases: an overview and some applications. Stat Med 1991; 10: 585–98. 2057657 10.1002/sim.4780100410

