# Supplementary material for: Place of death and other factors associated with unnatural mortality in patients with serious mental disorders: population-based retrospective cohort study
Source: BJPsych Open. 2019 Mar 4;5(2):e23. doi: 10.1192/bjo.2019.5 (PMC6401542; doi:10.1192/bjo.2019.5)
Supplement: Supplementary file 1 [file S205647241900005Xsup001.docx]

Supplementary Table 1: Descriptive statistics for the place of death of those patients that acutely accessed healthcare (A&E visits and/or hospital admissions) in the last month of life (N=510)

| Place of death (N=510) | N (%) | Cause of death, N (%) | |
| --- | --- | --- | --- |
| Care home | 22 (4.3) | Natural | 22 (100.0) |
|  |  | Unnatural | 0 |
| Home | 60 (11.8) | Natural | 54 (90.0) |
|  |  | Unnatural | 6 (10.0) |
| Hospice | 15 (2.9) | Natural | 15 (100.0) |
|  |  | Unnatural | 0 |
| Hospital | 404 (79.2) | Natural | 377 (93.3) |
|  |  | Unnatural | 27 (6.7) |
| Other | 9 (1.8) | Natural | 1 (11.1) |
|  |  | Unnatural | 8 (88.9) |
